# The function of Shp2 tyrosine phosphatase in the dispersal of acetylcholine receptor clusters

**DOI:** 10.1186/1471-2202-9-70

**Published:** 2008-07-23

**Authors:** Yueping K Qian, Ariel WS Chan, Raghavan Madhavan, H Benjamin Peng

**Affiliations:** 1 Department of Biology, Hong Kong University of Science and Technology, Clear Water Bay, Kowloon, Hong Kong, PR China

## Abstract

**Background:**

A crucial event in the development of the vertebrate neuromuscular junction (NMJ) is the postsynaptic enrichment of muscle acetylcholine (ACh) receptors (AChRs). This process involves two distinct steps: the local clustering of AChRs at synapses, which depends on the activation of the muscle-specific receptor tyrosine kinase MuSK by neural agrin, and the global dispersal of aneural or "pre-patterned" AChR aggregates, which is triggered by ACh or by synaptogenic stimuli. We and others have previously shown that tyrosine phosphatases, such as the SH2 domain-containing phosphatase Shp2, regulate AChR cluster formation in muscle cells, and that tyrosine phosphatases also mediate the dispersal of pre-patterned AChR clusters by synaptogenic stimuli, although the specific phosphatases involved in this latter step remain unknown.

**Results:**

Using an assay system that allows AChR cluster assembly and disassembly to be studied separately and quantitatively, we describe a previously unrecognized role of the tyrosine phosphatase Shp2 in AChR cluster disassembly. Shp2 was robustly expressed in embryonic Xenopus muscle in vivo and in cultured myotomal muscle cells, and treatment of the muscle cultures with an inhibitor of Shp2 (NSC-87877) blocked the dispersal of pre-patterned AChR clusters by synaptogenic stimuli. In contrast, over-expression in muscle cells of either wild-type or constitutively active Shp2 accelerated cluster dispersal. Significantly, forced expression in muscle of the Shp2-activator SIRPα1 (signal regulatory protein α1) also enhanced the disassembly of AChR clusters, whereas the expression of a truncated SIRPα1 mutant that suppresses Shp2 signaling inhibited cluster disassembly.

**Conclusion:**

Our results suggest that Shp2 activation by synaptogenic stimuli, through signaling intermediates such as SIRPα1, promotes the dispersal of pre-patterned AChR clusters to facilitate the selective accumulation of AChRs at developing NMJs.

## Background

Synapses facilitate efficient neuronal communication by bringing close together the organelles and molecules involved in the release and detection of neurotransmitters. Thus, to understand how synapses function, it is necessary to elucidate the signaling pathways that regulate the development of cellular specializations unique to synapses. For this purpose, one particular synaptic specialization – found at the vertebrate neuromuscular junction (NMJ) – has been examined extensively over the past several decades. Enriched in the postsynaptic domain of the NMJ are ion channels named acetylcholine (ACh) receptors (AChRs) that open upon binding to the neurotransmitter ACh; opening of AChRs depolarizes the synaptic region of muscle to trigger contraction. The synaptic aggregation of AChRs during NMJ assembly is therefore a process of paramount importance, one that is recognized to be tightly controlled by the combined actions of both muscle- and nerve-derived factors.

AChR clustering in muscle is initiated by the activation of the receptor tyrosine kinase MuSK (muscle-specific kinase) and it is mediated by the cytoplasmic protein rapsyn that binds to AChRs and tethers them to the cytoskeleton [1-3]. During development, rudimentary AChR clusters first appear before innervation in a MuSK-dependent manner and bestride the midline of embryonic muscle fibers [4,5]; these clusters are referred to as "pre-patterned" clusters because they form in the absence of neural influence. Later, in innervated muscle, AChRs become concentrated at incipient synapses where MuSK is locally stimulated by the nerve-derived factor agrin [4-6]. This synaptic accumulation of AChRs is furthermore coupled with the disassembly of the pre-patterned AChR clusters [7-10], and these two distinct processes together help generate a 1000-fold higher density of AChRs at the NMJ compared to extra-synaptic muscle [11].

The disassembly of pre-patterned AChR clusters, which is our focus here, was first shown to occur independently of AChR activity [7-10]; however, it was also reported that in rat and chick myotubes AChR agonists trigger the loss of AChR clusters [12,13], an issue that has recently attracted renewed attention. It has been observed that pre-patterned AChR aggregates, which are eliminated by embryonic day 17 or 18 (E17-18) in innervated muscle fibers of normal mice, are retained in the muscles of mutant mice with defective motor innervation [4,5] and of mice lacking the gene encoding the ACh biosynthetic enzyme choline acetyltransferase (CHAT) [14,15]. These findings have engendered the view that ACh directs the disassembly of AChR aggregates, and it has been proposed that neural agrin protects synaptic AChRs against ACh-induced dispersal [16]. The dispersal of AChR clusters by ACh is further thought to involve the ser/thr kinase Cdk5 [17,18] whose activity, in turn, appears to depend on the regulation of the protease calpain by rapsyn [19].

AChR activity-independent AChR dispersal has been well documented in frog and fish muscle where synaptogenic stimuli trigger both the assembly and disassembly of AChR aggregates [7,9,10,20-22]. In our studies we have used embryonic Xenopus muscle cultures to focus on the regulation of the AChR dispersal process by tyrosine phosphatases [23-25] because it is known that tyrosine phosphorylation stabilizes AChR clusters by strengthening links between AChRs and the actin cytoskeleton [26-31]. We showed that tyrosine phosphatases mediate the dispersal of pre-patterned AChR clusters by synaptogenic stimuli and that tyrosine dephosphorylation of pre-patterned clusters precedes the loss of AChRs from these sites in situ [2,23-25,32]. To date, however, the specific phosphatases involved in dispersing AChR clusters and the signaling pathways that stimulate them have remained unknown. These issues were addressed here using pharmacological and molecular methods and we describe for the first time a role of the tyrosine phosphatase Shp2, and its activator signal regulatory protein α1 (SIRPα1), in the disassembly of pre-patterned AChR clusters.

## Methods

### Reagents

Recombinant heparan-binding growth associated molecule (HB-GAM) was generously provided by Dr. Heikki Rauvala (University of Helsinki). Neural agrin was obtained as described [33]. The following reagents were purchased: NSC-87877 (Acros Organics; Fairlawn, NJ); tetramethylrhodamine-conjugated α-bungarotoxin (R-BTX) (Molecular Probes; Eugene, OR); anti-phosphotyrosine (mAb4G10) and anti-STEP tyrosine phosphatase monoclonal antibodies (Upstate Biotechnology; Lake Placid, NY); anti-Shp2 monoclonal antibody and protein tyrosine phosphatase antibody kit (BD Biosciences; San Jose, CA); secondary antibodies conjugated to FITC (Zymed; South San Francisco, CA) and horseradish-peroxidase (HRP) (Jackson Immuno Research Laboratories, Inc; West Grove, PA); Triton X-100 (TX-100) (Pierce; Rockford, IL).

### Whole-mount in situ hybridization

Xenopus embryos collected at different developmental stages were fixed in MEMFA solution (0.1 M MOPS, pH 7.4, with 2 mM EGTA, 1 mM MgSO4, and 3.7% formaldehyde) for 2 h at room temperature and then stored in absolute methanol at -20°C. Embryos were re-hydrated through a graded methanol series before carrying out in situ hybridization as described [34]. The cDNA encoding Xenopus SH2 domain-containing tyrosine phosphatase Shp2 in pCS2+ vector was kindly provided by Dr. Benjamin Neel (Beth Israel Deaconess Medical Center). This was used to synthesize digoxigenin-labeled cRNA probes in the antisense and sense directions with SP6 and T3 RNA polymerases with the Riboprobe In Vitro Transcription kit (Promega; Madison, WI). Hybridization reactions were carried out in parallel with these probes at a concentration of 0.1 μg/ml (in the presence of 1 mg/ml Torula RNA) overnight at ~60°C. Embryos were washed extensively with several buffers, including those containing RNaseA (20 μg/ml) and RNase T1 (10 U/ml), and then stained overnight at 4°C with alkaline phosphatase-linked anti-digoxigenin antibody. Chromogenic reaction was carried out by using NBT/BCIP as substrate in the alkaline phosphatase buffer (100 mM Tris HCL, 50 mM MgCl_2_, 100 mM NaCl, 0.1% Tween-20, 5 mM levamisol). Embryos were made transparent with a 2:1 mixture of benzyl benxoate:benzyl alcohol and imaged using a color CCD camera.

### Protein extract preparation and immuno-blotting

C2 mouse myoblasts (American Type Culture Collection) were differentiated into myotubes (4–5 d) and proteins were extracted from them using a TX-100 buffer (100 mM Tris, pH 7.4, 150 mM NaCl, 5 mM EDTA, 1% TX-100, and 1 mM Na-pervanadate; 1 ml buffer/10 cm dish) as described [25]. To extract Xenopus embryonic muscle proteins, dissected tadpole tails were homogenized in the above Triton buffer and the homogenates were incubated for 30 min on ice (with frequent mixing) before centrifuging them to obtain clarified extracts. Proteins were resolved by SDS-polyacrylamide gel electrophoresis and transferred to PVDF membranes, which were blocked in Tris-buffered saline containing 0.1% Tween-20 and 5% milk and immuno-blotted with antibodies diluted in the same blocking buffer. Primary antibodies were detected using appropriate HRP-linked secondary antibodies and enhanced chemiluminescence substrate (West Pico, Pierce).

### Shp2 and SIRPα1 cDNA constructs and mRNA synthesis

Human wild-type and mutant Shp2 cDNA constructs were from Dr. Benjamin Neel (Beth Israel Deaconess Medical Center). The mutant Shp2s were Shp2 E76A, which has a point mutation in the N-SH2 domain and produces a constitutively active Shp2 [35], and Shp2 deltaP, which has a deletion in the catalytic domain and, lacking both catalytic activity and substrate-binding ability, acts as a dominant-negative mutant [36]. Wild-type Shp2 (Shp2 WT), Shp2 E76A and Shp2 deltaP cDNAs were cloned into pSP64R1 vector [35]. Human full-length SIRPα1 (SIRP-FL) and truncated SIRPα1 (SIRP-TR) constructs were from Dr. David Clemmons (University of North Carolina at Chapel Hill). In SIRP-TR, the Y475 site of the tandem phosphorylation domain of SIRPα1 that binds to Shp2 is eliminated, generating a SIRPα1 mutant that does not activate Shp2 and functions as a dominant-negative suppressor of Shp2 signaling [37]. SIRPα1 sequences were in pcDNA3.1 vector [37] as were those encoding the reporter green fluorescent protein (GFP). Shp2 mRNAs were prepared with the SP6 in vitro transcription kit purchased from Ambion (Austin, TX) and the SIRPα1 and GFP mRNAs were prepared with the T7 ULTRA kit from the same company. Shp2 and SIRPα1 mRNAs were mixed with GFP mRNA before microinjection.

### Xenopus embryo microinjection and preparation of primary muscle cultures

Mixtures of Shp2 or SIRPα1 and GFP mRNAs (total 10 ng or less, in a volume of 4.6–9.2 nl) were injected into one cell of 2–4 cell stage Xenopus embryos with a Drummond Nanoject oocyte injector (Drummond Scientific Co., Broomall, PA). Injected embryos were maintained in Holfreter's solution containing 4% Ficoll and those expressing exogenous mRNAs were identified by green fluorescence and used for preparing muscle cultures [38]. Myotomal muscle cells were cultured from stage 20–22 embryos as described [39] and plated on glass coverslips coated with ECL (entactin-collagen IV-laminin) substrate (Upstate Biotechnology). AChR redistribution experiments used muscle cells maintained in culture for 2–6 days.

### Shp2-inhibitor, R-BTX/antibody labeling and microscopy

The Shp2-inhibitor NSC-87877 [40] was dissolved in water and then diluted into culture medium as required. R-BTX-labeled muscle cells were incubated in culture medium without or with NSC-87877, in the absence or presence of agrin or HB-GAM beads. Polystyrene beads (10 μm; Polysciences, Warrington, PA) were coated with HB-GAM as described [41]. In cases where muscle cells expressed exogenous mRNAs, live cultures were used to examine cells of comparable GFP fluorescence intensity and AChR cluster redistribution data were collected from a minimum of five separate mRNA injections and culture preparations. In experiments using wild-type cultures, muscle cells were fixed with cold 95% ethanol and stained with primary and fluorescent secondary antibodies. Labeled cells were imaged using an Olympus IX70 microscope equipped with a Hamamatsu ORCAII cooled-CCD or ORCA-ER camera controlled by MetaMorph software (Universal Imaging, West Chester, PA).

## Results

### Shp2 expression in Xenopus embryonic muscle

**Figure 1:**
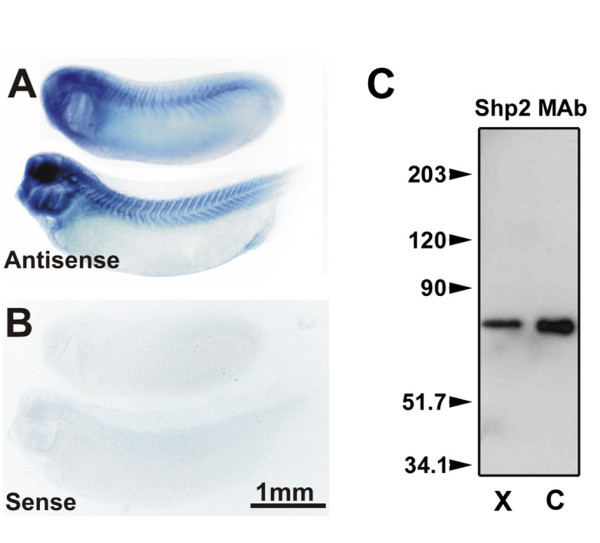
**Shp2 expression in embryonic Xenopus muscle**. In situ hybridization assays for Shp2 mRNA were carried out with anti-sense (A) and control sense (B) probes using stage 22 (A and B, upper) and 28 (lower) Xenopus embryos. Specific labeling was observed with Shp2 mRNA anti-sense probe in myotomal muscle and brain. C. TX-100 total protein extracts of Xenopus tadpole tail muscle ("X") and cultured C2 myotubes ("C") were immunoblotted with an anti-Shp2 monoclonal antibody. A band of about 70 kD was observed in both samples. Positions of molecular weight markers are indicated on the left.

We previously reported that Shp2, a tyrosine phosphatase with two SH2 domains [42], modulates AChR clustering in C2 mouse myotubes by suppressing agrin/MuSK signaling [25,43], but whether or not Shp2 (or a different phosphatase) participates in the dispersal of pre-patterned AChR clusters by synaptogenic stimuli could not be tested. This is because in the C2 myotube cultures very few pre-patterned AChR clusters developed and those that did form resembled new clusters induced by agrin. Thus, to investigate tyrosine phosphatase involvement specifically in the disassembly of AChR clusters, here we used primary cultures of embryonic Xenopus muscle cells in which the dispersal of pre-patterned AChR clusters by synaptogenic stimuli can be examined separately from the induction of new AChR clusters and both processes can be quantified (see below) [23-25]. We began by staining the myotomal muscle cell cultures with antibodies against twelve different tyrosine phosphatases and found that an anti-Shp2 antibody, unlike other antibodies tested, labeled the cells strongly and uniformly (unpublished observations), which led us to determine if Shp2 was present in embryonic Xenopus muscle in vivo. Whole mount in situ hybridization was carried out with an antisense digoxigenin-labeled RNA probe made from Xenopus Shp2 cDNA, and the results showed that Shp2 mRNA was enriched in the brains and myotomes of embryos at stages 22 and 28 (Figure 1A). The detection of Shp2 mRNA was specific as no hybridization occurred with a control sense probe (panel B). Moreover, in immuno-blotting assays using tadpole tail extracts, an anti-Shp2 monoclonal antibody stained a single protein band of the size expected for Shp2 (~70 kD), and the antibody also stained a band of similar size in C2 mouse myotube extracts (Figure 1C). These results combined with previous demonstrations of Shp2 expression in rodent muscle cells [25,44] identified Shp2 as a candidate tyrosine phosphatase involved in the dispersal of pre-patterned AChR clusters.

### Shp2 inhibition and AChR cluster dispersal

**Figure 2:**
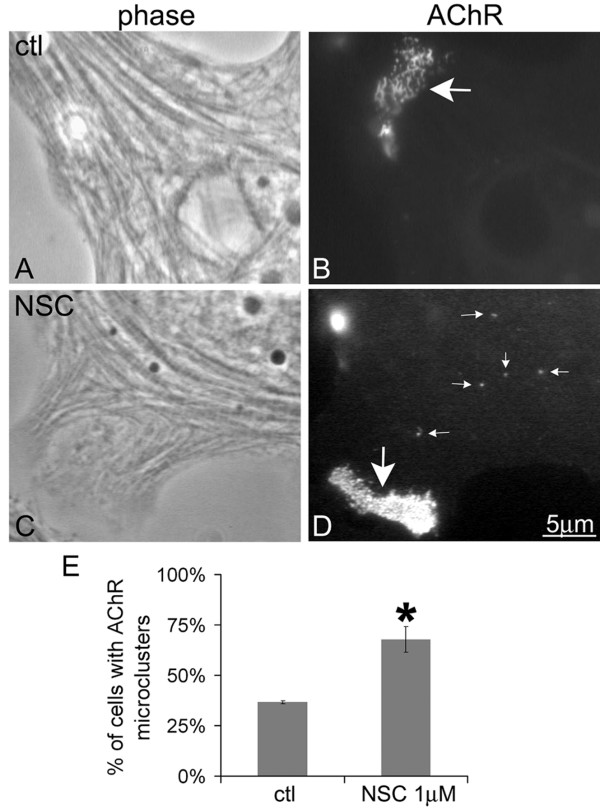
**Effect of Shp2 inhibitor NSC-87877 on AChR micro-cluster formation**. Cultured Xenopus muscle cells, used here and in all experiments described below, were labeled with R-BTX and then incubated overnight in control culture medium (A, B) or in medium containing 1 μM NSC-87877 (NSC) (C, D). Large pre-patterned AChR clusters, often >10 μm in width, were detected in both control and NSC-treated cells (B, D; large arrows), but in cells exposed to NSC, AChR micro-clusters were also frequently observed (D; small arrows). E. Quantification of pooled data from multiple experiments revealed that AChR micro-clusters developed in nearly twice the number of muscle cells incubated in 1 μM NSC-87877 as those maintained in control medium. Mean and SEM values are shown here and in all following figures; control cells, n = 103; NSC-treated cells, n = 109; t-test *p < 0.005.

To test whether Shp2 activity influences the dispersal of pre-patterned AChR clusters during NMJ formation, we exposed cultured Xenopus muscle cells to NSC-87877, a recently identified chemical inhibitor of Shp2 [40]. Cells were stained with rhodamine-conjugated α-bungarotoxin (R-BTX) to label AChRs and incubated overnight without or with the drug. First, in the absence of synaptogenic stimulation, small AChR clusters (0.5–2 μm in diameter) developed in many of the NSC-treated but not control cells (Figure 2A–D); normal, pre-patterned clusters (see below) were detected in both NSC-treated and control cells. When used at 1 μM, NSC-87877 nearly doubled the percentage of muscle cells with AChR micro-clusters (Figure 2E), and at higher concentrations of NSC (10 μM or more) there appeared to be a further increase (~50%) in AChR clustering (data not shown). In the 0.1–2 μM range, NSC-87877 is likely to inhibit (other than Shp2) Shp1 (IC50 ~0.3 μM) and to a lesser extent PTP1B (IC50 ~1.7 μM), but at high concentrations other phosphatases could be affected [40]. As no significant staining of Xenopus muscle cells was detected using either the anti-Shp1 or anti-PTP1B antibody, it is possible that at a concentration of 1 μM NSC's primary target in these cells is Shp2. Moreover, unlike NSC, several control drugs against proteins unrelated to Shp2 had no effect on AChR clustering (unpublished observations) [25,32].

The above results and those showing enhanced AChR clustering in C2 mouse myotubes following the depletion or inhibition of Shp2 [25,43] support the conclusion that in Xenopus muscle cells, as in mouse myotubes, Shp2 limits the formation of AChR clusters. But does Shp2 also mediate the dispersal of pre-patterned AChR clusters? To answer this we again used NSC-treatment and examined the AChR clusters that form spontaneously in Xenopus muscle cells that have been in culture for 2 or more days. These large AChR clusters, which are often more than 10–15 μm across and were previously referred as "hot spots", were the ones considered here to be "pre-patterned" because they develop without synaptogenic stimulation. These pre-patterned clusters are stable for several days and do not disperse on their own, but they are rapidly disassembled when muscle cells are innervated by co-cultured nerves or when the cells are exposed to agrin or growth factor-coated beads [23-25]. We have previously demonstrated that the rate of dispersal of a pre-patterned cluster is directly related to its distance from a synaptogenic stimulation site as well as to the strength of the external stimulus [10,23]. In all experiments described below, muscle cells were labeled with R-BTX before synaptogenic stimulation so that the true disassembly of pre-patterned clusters could be followed, and, in all cases, untreated (control) cells from the same culture preparation were examined in parallel to confirm that pre-patterned clusters had developed normally. These control cells additionally showed that during the course of the assays (none of which lasted longer than one day) there was no significant increase in spontaneous AChR clustering, which is in accord with our previous work [10]. This point is important to consider because the rapid, net decrease in the number of pre-patterned AChR clusters that follows synaptogenic stimulation demonstrates that external stimuli actively disperse old AChRs clusters rather than merely preventing (if this occurs at all) the assembly of additional, new pre-patterned clusters (leaving the original pre-patterned clusters intact). Indeed, we have previously used growth factor-coated beads and time-lapse imaging of identified pre-patterned clusters to show their dispersal by applied stimuli in live cells [23]. Lastly, in this study pure muscle cultures were used throughout to avoid any (currently unknown) presynaptic effect of NSC-87877 that could potentially influence AChR redistribution in nerve-muscle co-cultures.

**Figure 3:**
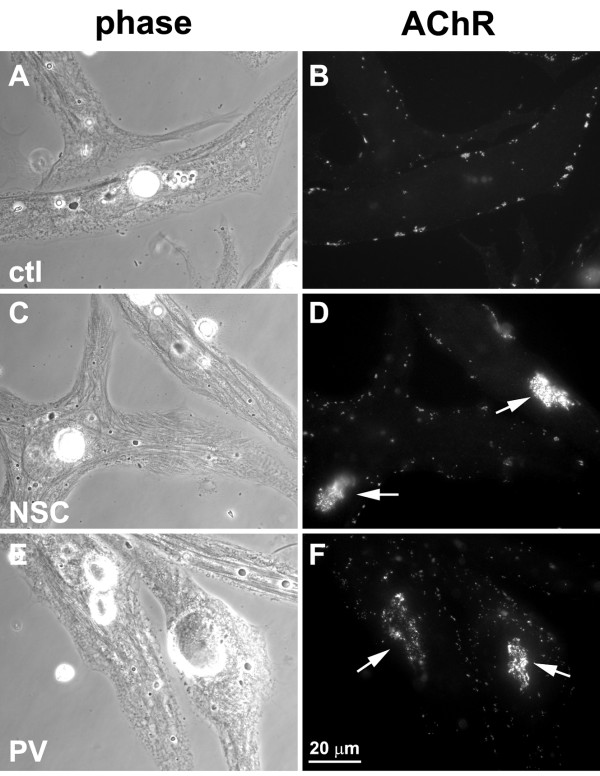
**Effect of NSC-87877 on agrin-induced dispersal of pre-patterned AChR clusters**. Muscle cells labeled with R-BTX were incubated overnight in culture medium containing agrin (ctl; A, B), agrin plus 2 μM NSC-87877 (NSC; C, D), or agrin plus 20 μM pervanadate (PV; E, F). In agrin-treated muscle cells AChR micro-clusters (~0.5–2 mm in diameter) were found but larger clusters were not generally detected (B). However, in cells incubated with agrin and NSC (D) or agrin and PV (F), both micro-clusters and large pre-patterned clusters were present (arrows).

In R-BTX-labeled muscle cells, overnight treatment with agrin generated AChR micro-clusters (~0.5–2 μm) and triggered the loss of pre-patterned clusters (Figure 3A–B) as described before [25,33]; in the absence of agrin pre-patterned clusters remained unaffected (as in Fig. 2; control). In contrast, in muscle cells exposed to agrin plus NSC-87877, both small AChR clusters and the larger aggregates resembling pre-patterned AChR clusters were detected (panels C-D). Similarly, small and large AChR clusters were present on the surface of muscle cells treated with agrin and the tyrosine phosphatase inhibitor pervanadate (panels E-F) as shown previously [23-25]. These results suggested that agrin-dependent dispersal of pre-patterned AChR clusters was blocked by the inhibition of Shp2.

**Figure 4:**
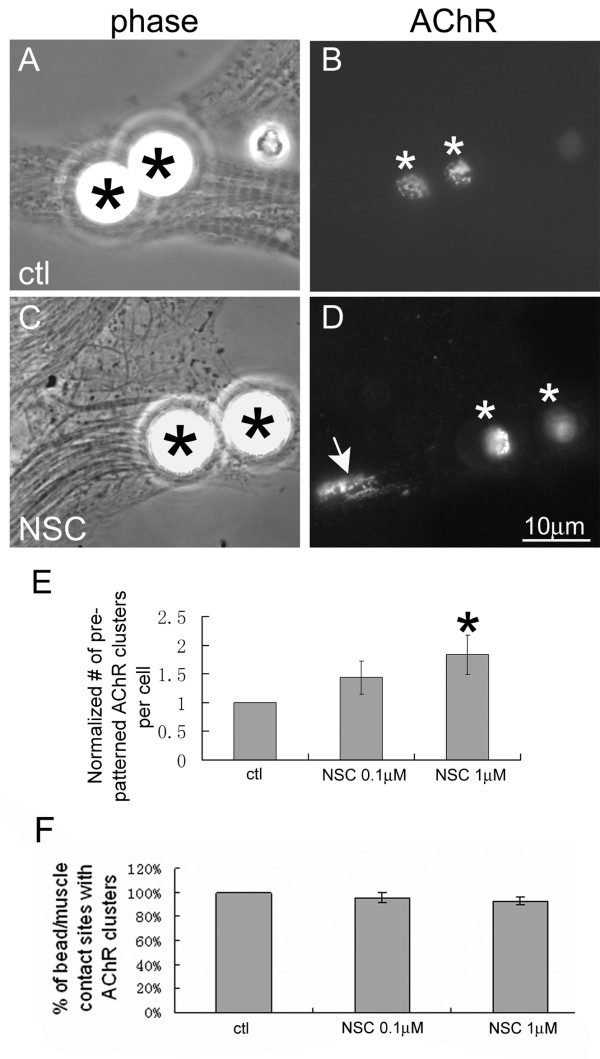
**Inhibition of HB-GAM bead-induced dispersal of pre-patterned AChR clusters by NSC-87877**. R-BTX-labeled muscle cells were stimulated overnight with HB-GAM beads in the absence (A, B) or presence of 1 μM NSC-87877 (C, D). In control cells only bead-induced AChR clusters were present (B; asterisks) but in the NSC-treated cells both bead-induced (D; asterisks) and pre-patterned AChR clusters (arrows) were detected. The number of pre-patterned AChR clusters in bead-stimulated cells and the fraction of beads that induced new AChR clusters were quantified from multiple experiments using 0.1 or 1 μM NSC and the results were normalized relative to cells maintained in control medium (E, F). NSC-treatment inhibited the dispersal of pre-patterned AChR clusters but did not significantly affect the induction of new AChR clusters by HB-GAM beads. Control, n = 95; 0.1 μM NSC, n = 115; 1 μM NSC, n = 129; *p < 0.05, 1 μM NSC relative to control.

To accurately quantify NSC's effect on dispersal, we stimulated muscle cells with beads coated with heparan-binding growth-associated molecule (HB-GAM), which induce clusters of AChRs associated with proteins that are also found at nerve-muscle synapses and AChR clusters generated by agrin [2,24]. These beads generate AChR clusters only at sites where they directly contact muscle cells, which eliminates any chance of mistaking induced AChR clusters for pre-patterned ones [23,25,41], and the ability to count separately these dissimilar (bead-induced and pre-patterned) clusters makes it possible to easily distinguish an increase of spontaneous AChR clustering caused by a drug-treatment (perhaps by disinhibition of cluster formation) from the inhibition of cluster disassembly (see below). Following overnight stimulation with HB-GAM beads, AChR clusters were found at bead-muscle contacts in both control and NSC-treated cells, but unlike in control cells, in NSC-treated cells pre-patterned AChR clusters were also readily detected (Figure 4A–D). We determined the percentage of beads that induced new AChR clusters and counted the pre-patterned AChR clusters that were present on cells after exposure to beads. The latter numbers were then normalized relative to those obtained from untreated and NSC-treated cells examined in parallel to which beads had not been added – this was done to compensate for any potential change (however small) in the number of pre-patterned clusters that might have arisen during the assay period. Our pooled data showed that NSC-treatment inhibited bead-stimulated dispersal of pre-patterned AChR clusters (Figure 4E) but not the induction of new AChR clusters (panel F). These results also indicated that cluster dispersal was not blocked due to an inability of the beads to initiate signaling per se in the presence of the Shp2-inhibitor. This selective inhibition of dispersal by NSC-addition is akin to what is observed when muscle cells are exposed to pervanadate, but it differs from the effects of other compounds that either do not influence dispersal or which suppress dispersal by blocking synaptogenic signaling [23,25,32,45].

**Figure 5:**
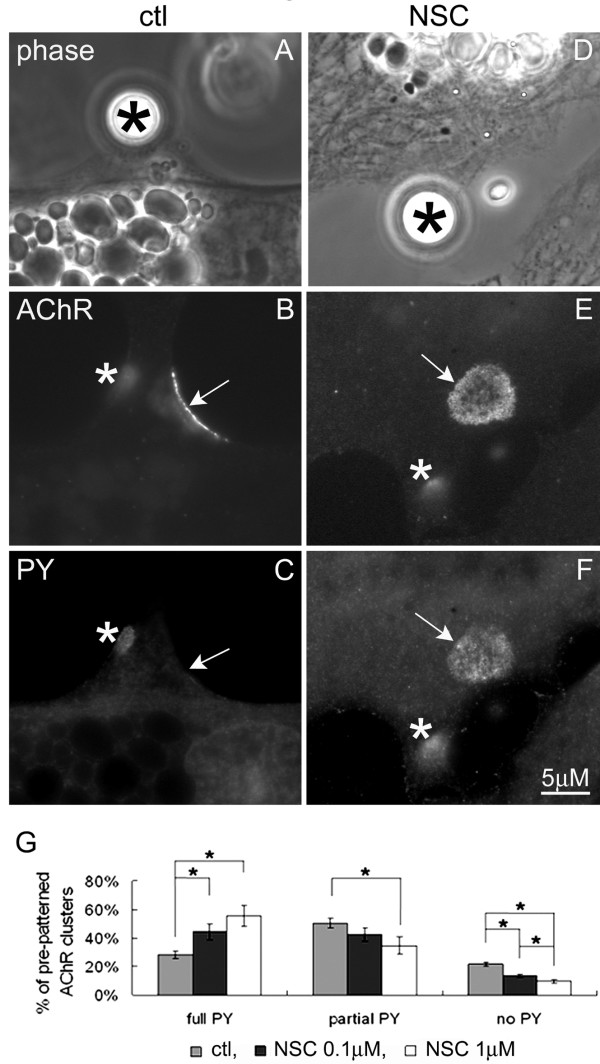
**Blockage of tyrosine dephosphorylation of pre-patterned AChR clusters by NSC-treatment**. R-BTX-labeled muscle cells were exposed to HB-GAM beads for 4 h in the absence (A-C) or presence of 1 μM NSC-87877 (D-F), fixed and then stained with anti-phosphotyrosine (mAb4G10) and FITC-conjugated secondary antibodies. In the example of the control muscle cell shown here, phosphotyrosine is detected at the bead-induced cluster (B, C; asterisks) but not at the pre-patterned cluster (B, C; arrow), but in the NSC-treated muscle cell, both the bead-induced cluster (E, F: asterisks) and the pre-patterned cluster (E, F: arrows) contain phosphotyrosine. G. Pooled data from multiple experiments were quantified after sorting the pre-patterned AChR clusters present on bead-stimulated cells as being fully tyrosine phosphorylated or partially or fully dephosphorylated. Control, n = 127; 0.1 μM NSC, n = 129; 1 μM NSC, n = 94; *p < 0.05 NSC relative to control.

To directly evaluate NSC-87877's effect on the AChR cluster disassembly process, muscle cells were stimulated with HB-GAM beads for 4 h and stained with a monoclonal antibody against phosphotyrosine (mAb4G10). Because the loss of phosphotyrosine labeling precedes the depletion of AChRs from moribund pre-patterned clusters, the phosphorylation state of AChR-containing clusters (i.e., the fractional area of a pre-patterned AChR cluster labeled by anti-phosphotyrosine) can be used to measure the spread of the dispersal signal [25]. When we examined the phosphorylation of pre-patterned AChR clusters in muscle cells in which beads had induced new AChR clusters, the pre-patterned clusters in control cells were found to be tyrosine dephosphorylated significantly more (Figure 5A–C) than those in NSC-treated cells (panels D-F). In the examples shown (Fig. 5), the pre-patterned AChR cluster in the control muscle cell is devoid of phosphotyrosine whereas the one in the NSC-treated cell has lost but a tithe of its phosphotyrosine. Pooled data were quantified by sorting pre-patterned AChR clusters in bead-stimulated cells (both control and NSC-treated) as being fully tyrosine phosphorylated, partially dephosphorylated (where labeling for phosphotyrosine overlapped with less than half of that for AChRs) and completely dephosphorylated (where no phosphotyrosine enrichment was detected at AChR clusters). These results indicated that Shp2-inhibition hindered the spread of the AChR cluster dispersing signal (panel G).

The above NSC-87877 data suggest that Shp2 signaling promotes the disassembly of pre-patterned AChR clusters, which agrees with and extends our earlier finding that tyrosine phosphatase activity mediates AChR cluster dispersal [2,23,25].

### Exogenous Shp2 expression and AChR cluster dispersal

**Figure 6:**
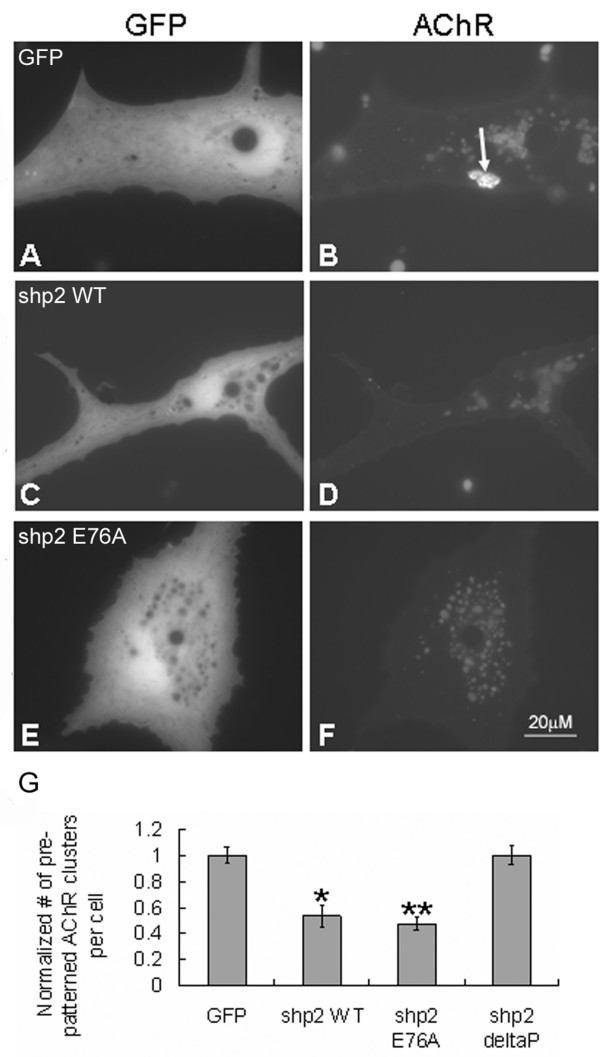
**Effects of over-expression of wild-type or constitutively active Shp2 on pre-patterned AChR cluster formation**. Wild-type and mutant Shp2 proteins were over-expressed in Xenopus muscle cells by mRNA injection. Muscle cells expressing green fluorescent protein alone (GFP; A, B) or GFP together with wild-type Shp2 (Shp2 WT; C, D), constitutively active Shp2 (Shp2 E76A; E, F) or dominant-negative Shp2 (Shp2 deltaP; images not shown) were stained with R-BTX (B, D, F) to visualize pre-patterned AChR clusters. In muscle cells expressing GFP, normal pre-patterned AChR clusters were found (B; arrow), but in cells with wild-type or constitutively active Shp2 fewer clusters were detected (D, F). G. Data from multiple micro-injection experiments were quantified and the numbers of pre-patterned clusters in cells expressing wild-type and active Shp2 were normalized relative to the number obtained from GFP-expressing cells. GFP-cells, n = 356; Shp2 WT, n = 98; Shp2 E76A, n = 327; Shp2 deltaP, n = 356; *p < 0.05; **p < 0.001 relative to GFP-cells.

**Figure 7:**
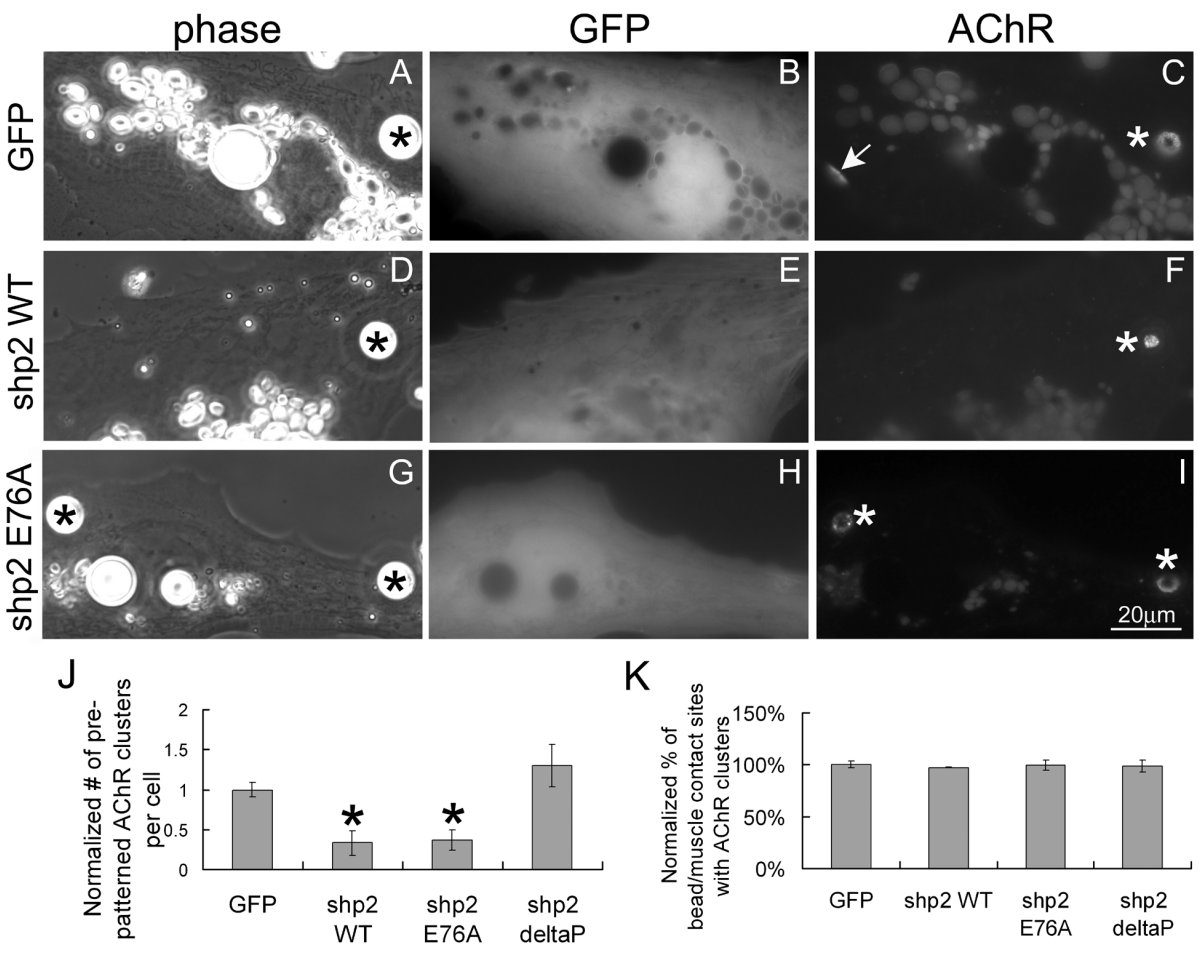
**Acceleration in the dispersal of pre-patterned AChR clusters resulting from exogenous wild-type or constitutively active Shp2 expression**. R-BTX labeled muscle cells expressing GFP only (A-C), or GFP plus Shp2 WT (D-F), Shp2 E76A (E-G) or Shp2 deltaP (not shown) were stimulated overnight with HB-GAM beads. In a fraction of bead-stimulated cells expressing GFP alone, pre-patterned AChR clusters were still present, but the fraction of such cells expressing wild-type or active Shp2 that retained pre-patterned clusters was significantly lower. To quantify these results, the number of pre-patterned clusters counted in bead-stimulated cells was divided by the number obtained from muscle cells that had not been exposed to beads (to offset differences in pre-patterned cluster formation; see text). Results normalized relative to GFP-cells are shown in panel J. GFP-cells, n = 212; Shp2 WT, n = 60; Shp2 E76A, n = 138; Shp2 deltaP, n = 127; *p < 0.01; **p < 0.05. Panel K shows that the expression of Shp2 proteins did not affect bead-induced AChR clustering.

To investigate Shp2's influence on AChR cluster dispersal using a different approach, we next examined muscle cells expressing exogenous Shp2 proteins. For this, mRNAs encoding wild-type Shp2 (Shp2 WT), constitutively active Shp2 (Shp2 E76A) or inactive Shp2 (Shp2 deltaP) and the reporter green fluorescent protein (GFP) were injected into Xenopus embryos from which muscle cells were cultured. AChRs were labeled with R-BTX and cells with exogenous proteins were identified by green fluorescence. In muscle cells expressing wild-type or constitutively active Shp2, fewer pre-patterned AChR clusters were found than in cells expressing GFP alone (Figure 6A–F; quantified in panel G), suggesting that Shp2 inhibited the formation of AChR clusters in these muscle cells as in C2 myotubes [25,43]. Moreover, after stimulation with HB-GAM beads, more complete dispersal of pre-patterned AChR clusters occurred in muscle cells expressing wild-type or constitutively active Shp2 compared to those expressing GFP only (Figure 7A–I). To quantify these data, we divided the number of pre-patterned AChR clusters in bead-stimulated cells (expressing GFP or GFP plus Shp2 proteins) by the number of pre-patterned clusters in cells not exposed to beads (as described in the previous section); here, this compensated for the reduced formation of pre-patterned AChR clusters in cells over-expressing Shp2. These ratios were then further normalized using those calculated for cells expressing GFP alone. These results indicated that the presence of excess wild-type or constitutively active Shp2 proteins in muscle cells is sufficient for promoting AChR cluster dispersal (Figure 7J). In these assays the exogenous Shp2 proteins did not block the induction of new AChR clusters (panel K) suggesting that higher Shp2 activity and/or the activity of additional tyrosine phosphatases is required to inhibit AChR clustering by HB-GAM beads.

Here we also tested whether ectopic expression of the inactive Shp2 in Xenopus muscle cells would significantly hinder AChR cluster dispersal and found that not to be the case (images not shown; see quantified data in Figures 6, 7). This is possibly because we obtained only low-level expression of inactive Shp2 in cells (as indicated by GFP fluorescence) and attempts to enhance this mutant's expression by injecting more of its mRNA into embryos disrupted development as previously noted [35,36]. That Shp2 signaling plays a role in mediating the dispersal of AChR clusters, however, is suggested by experiments described below using the Shp2-activator SIRPα1 and a mutant form of it.

### SIRPα1 over-expression and AChR cluster dispersal

**Figure 8:**
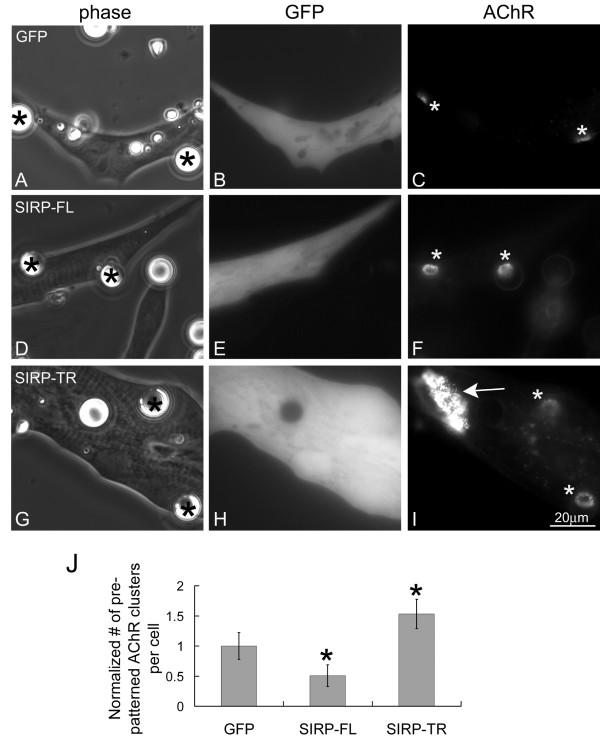
**SIRPα1's regulation of pre-patterned AChR cluster dispersal**. R-BTX labeled muscle cells expressing GFP alone (A-C) or GFP and wild-type SIRPα1 (SIRP-FL; D-F) or dominant-negative SIRPα1 (SIRP-TR, G-I) were exposed to HB-GAM beads overnight. In the muscle cell expressing dominant-negative SIRPα1, a large pre-patterned AChR cluster was detected (I; arrow) in addition to the bead-induced clusters, which were present in control and Shp2-expressing cells (C, F, I; asterisks). Results, quantified as described in Fig. 7, revealed that the expression of wild-type SIRPα1 promoted the dispersal of pre-patterned AChR clusters whereas expression of dominant-negative SIRPα1 inhibited dispersal (panel J). GFP-cells, n = 198; SIRP-FL, n = 156; SIRP-TR, n = 169; *p < 0.05 relative to GFP-cells.

SIRPα1 is a transmembrane protein that, when tyrosine-phosphorylated, binds to Shp2's tandem SH2 domains and stimulates Shp2 activity [42]. We recently showed that in C2 mouse myotubes SIRPα1's tyrosine phosphorylation and Shp2-binding were enhanced by agrin-treatment and that increased SIRPα1/Shp2 signaling inhibited AChR cluster formation [43]. To test whether SIRPα1/Shp2 signaling also regulates AChR cluster dispersal, we injected Xenopus embryos with mRNAs encoding full-length SIRPα1 (SIRP-FL), which can stimulate Shp2, or truncated SIRPα1 (SIRP-TR), which lacks the Shp2-binding tyrosine phosphorylation domain and acts as a dominant-negative suppressor of Shp2 signaling in cells [37]. Muscle cells expressing GFP or GFP and the SIRPα1 proteins were exposed to HB-GAM beads and the number of pre-patterned AChR clusters remaining after stimulation was determined, divided by the number obtained from the cells not exposed to beads, and normalized relative to the value calculated for GFP-cells (as discussed above). Compared to the expression of GFP only (Figure 8A–C), expression of full-length SIRPα1 in muscle cells significantly enhanced pre-patterned AChR cluster dispersal by beads (panels D-F) whereas the expression of truncated SIRPα1 inhibited dispersal (panels G-I). These effects, quantified in panel J, were produced specifically by SIRPα1: in contrast to full-length SIRPα1, expression of a different phosphoprotein, p120 catenin (p120ctn), did not promote AChR cluster dispersal, and unlike truncated SIRPα1, phospho-mutant p120ctn did not block AChR redistribution [38]. Taken together with our Shp2 results, these findings suggest that full-length SIRPα1 accelerated AChR cluster dispersal by activating endogenous Shp2 and that truncated SIRPα1 suppressed dispersal by reducing the ability of endogenous signaling intermediates (SIRPα1 or related proteins) to activate Shp2 in response to synaptogenic stimuli.

## Discussion

The disassembly of pre-patterned AChR clusters that occurs in muscle following motor innervation facilitates selective AChR concentration at the NMJ. Here we studied the process by which AChR clusters are disassembled by synaptogenic stimuli using primary cultures of Xenopus muscle cells. We found that AChR cluster dispersal was expedited when active or activatable Shp2, or the Shp2-activator SIRPα1, was introduced into muscle cells, but that cluster dispersal was blocked when cells were exposed to an inhibitor of Shp2 or when they over-expressed a mutant form of SIRPα1 that curbs Shp2 signaling. These results suggest that Shp2 and its regulators such as SIRPα1 promote the disassembly of pre-patterned AChR clusters by synaptogenic stimuli during NMJ development.

Innervation of muscle triggers AChR concentration at the NMJ. For this to occur, three proteins are indispensable: rapsyn, for clustering AChRs [46], MuSK, for initiating the AChR aggregation process [47], and agrin, for enabling synaptic AChR accumulation [16,48]. AChR clusters are further stabilized by tyrosine kinases [29,30,49] and the dystrophin complex proteins [50] that directly or indirectly strengthen AChR-cytoskeleton linkage. Moreover, AChR clustering requires dynamic actin polymerization [45], which is regulated by small GTPases, their effectors and other modulators [51-54], and clustering is also promoted by a transmembrane protein, LRP4 [55], and the MuSK-binder, dok-7 [56].

In addition to AChR aggregation, innervation triggers the dispersal of pre-patterned AChR clusters. In Xenopus primary muscle cultures these two processes can be readily distinguished from one another and the redistribution of AChRs in response to various stimuli can be accurately quantified [23,25,32,41]. We have previously shown that tyrosine phosphatases mediate AChR cluster dispersal [23,25] and here we identified Shp2 as one of the phosphatases that facilitates dispersal. Treatment of muscle cells with NSC-87877, a selective Shp2 antagonist [40], hindered the dispersal of pre-patterned AChR clusters without preventing the induction of new AChR clusters, as also seen with the general phosphatase inhibitor pervanadate but not other drugs [23,25,32,45].

NSC-treatment enhanced AChR clustering in Xenopus muscle cells whereas the expression of active Shp2 had the opposite effect, consistent with our results in C2 myotubes [43]. In C2 myotubes, however, the expression of dominant-negative Shp2 promoted AChR cluster formation [43] but here no significant effect of this mutant on cluster induction was detected. Additionally, because the inactive Shp2 mutant was only poorly expressed in Xenopus muscle cells, we could not test whether "dominant-negative" inhibition of Shp2 would block AChR cluster dispersal, as suggested by our pharmacological assays. But because Shp2-dependent suppression of AChR clustering in C2 myotubes could be stimulated by the phosphoprotein SIRPα1 [43], we examined if exogenous SIRPα1 proteins can affect the dispersal of pre-patterned AChR clusters in Xenopus muscle cells. Expression of full-length and truncated SIRPα1, which enhance and inhibit Shp2 signaling [37], promoted and blocked AChR cluster dispersal, respectively. These results suggest that SIRPα1 (or a protein related to it) spreads the signal that globally activates Shp2 to facilitate AChR cluster disassembly.

**Figure 9:**
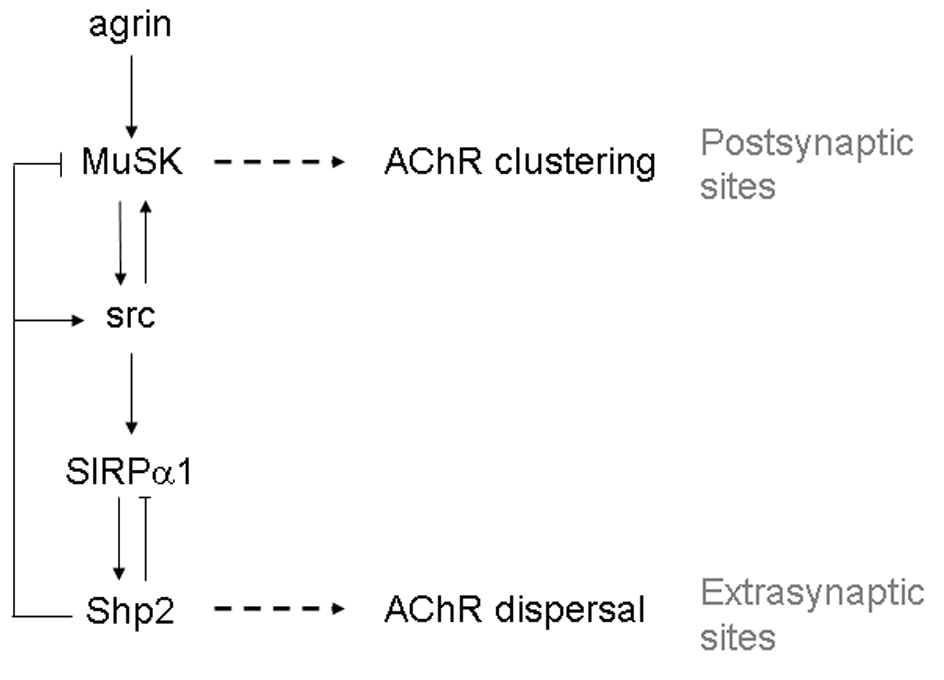
**A model for Shp2-dependent regulation of AChR redistribution**. Synaptogenic stimuli initiate two SIRPα1/Shp2-involving signaling cascades. Agrin/MuSK stimulates src family tyrosine kinases that, in addition to phosphorylating MuSK, phosphorylate SIRPα1 to activate Shp2, which then dephosphorylates (on tyr527) and stimulates more src, and thus more SIRPα1/Shp2, etc., to propagate the dispersal signal that disassembles pre-patterned AChR clusters at extrasynaptic sites. Locally at postsynaptic sites, SIRPα1/Shp2 signaling triggered by MuSK/src acts through a feedback loop to restrain the MuSK-initiated AChR clustering signal, which helps generate new AChR clusters selectively at postsynaptic sites. This form of regenerative signaling through src, SIRPα1 and Shp2 also has built-in self-regulation because Shp2 can dephosphorylate SIRPα1 to control its own activation state.

While our results have revealed a novel role of SIRPα1 and Shp2 in AChR dispersal, it is likely that other, as yet unidentified proteins are also involved. This is because inhibition of all tyrosine phosphatases with pervanadate blocks AChR cluster disassembly more effectively [23] than reagents that target SIRPα1 and Shp2. However, our current and previous findings suggest a signaling pathway for SIRPα1/Shp2-dependent dispersal of AChR clusters in response to a synaptogenic stimulus. Near innervation sites, for example, Shp2 activated by SIRPα1 in response to agrin-stimulation of MuSK could, in addition to curtailing the AChR clustering signal [25,43], dephosphorylate and increase the activity of src kinases [57] in the surrounding area to trigger more SIRPα1 phosphorylation and Shp2 activation, and so on (Figure 9). Such a "regenerative" signaling pathway with src, SIRPα1, and Shp2 functioning sequentially was described in integrin signaling [58] where it was shown that phosphorylation of SIRPα1 leads to the activation of Shp2, which stimulates src that phosphorylates SIRPα1. Because AChR clusters induced in muscle cells by growth factor-coated beads and agrin contain a similar complement of signaling and scaffolding proteins [2,24], it is possible that many of the same molecules activated following direct or indirect MuSK stimulation regulate the src, SIRPα1 and Shp2 signaling cascade. A spread of this signaling across muscle could trigger Shp2 to dephosphorylate AChR-associated proteins at pre-patterned clusters or AChRs themselves, which are in vitro substrates of Shp2 [59]; dephosphorylation of AChRs and/or other denizens of the pre-patterned clusters could then de-link AChRs from the cytoskeleton [31] and allow their redistribution.

Several studies (in addition to ours) have demonstrated that pre-patterned AChR cluster disassembly occurs in amphibian and fish muscle independently of AChR activity. For example, in Xenopus muscle cells, pre-patterned clusters are dispersed by innervation when all AChR channel activity is blocked using bungarotoxin or curare and no electrical activity or twitching is detected in muscle [7,9,20]. Moreover, during NMJ formation in vivo in zebrafish, pre-patterned AChR clusters are redistributed when saturating concentrations of bungarotoxin are used or even when the bungarotoxin is added together with inhibitors of sodium channels to further ensure blockade of all neuronal activity [22]. Thus, in these muscle cells, ACh-dependent AChR channel opening appears to be unnecessary for redistributing pre-patterned AChR aggregates during NMJ formation. Whether ACh can subtly influence the effectiveness with which other synaptogenic stimuli disperse pre-patterned clusters, however, requires further investigation.

In contrast to the above findings, in rodent and chick myotubes AChR agonists reduce surface AChR clusters [12,13] and muscle electrical activity suppresses extra-synaptic AChR synthesis [60,61]. Other studies have revealed a more direct role of ACh in AChR cluster disassembly, as summarized by this recent model [19]: ACh elevates muscle intracellular Ca^2+ ^and stimulates the protease calpain, which cleaves a protein named p35 to generate a p25 fragment [19]; p25 potently activates the protein kinase Cdk5, which disperses AChR clusters [17-19]. At the NMJ agrin promotes calpain's interaction with rapsyn, which inhibits calpain and suppresses p25 production to locally limit Cdk5 activity and block AChR dispersal [19]. Thus agrin is thought to protect synaptic AChRs against dispersal by ACh [16]. However, as discussed in these studies, some aspects of this dispersal pathway warrant further investigation. For example, calpain is activated in muscle more rapidly than Cdk5, suggesting that additional unknown factors regulate Cdk5 during dispersal [19]. Since Cdk5 phosphorylation sites have not been found in AChR subunits, it is also unclear exactly how Cdk5 disperses AChR clusters [18]. Intriguingly, AChR activity, which favors dispersal in embryonic muscle, stabilizes AChRs during synaptic competition [62], which could be due to changes in the molecular makeup of AChR subunits or clusters during development [18]. And, the elevation of intracellular Ca^2+ ^by agonists of AChRs and L-type calcium channels actually promote AChR clustering [63,64], suggesting that Ca^2+ ^can either favor AChR cluster assembly and stabilization or cluster destabilization and dispersal (through calpain/Cdk5).

Roles of Shp2 or other tyrosine phosphatases in ACh-dependent AChR dispersal have not been described, but phosphatase inhibition blocks ACh-independent dispersal caused by defects in src signaling [29,65,66] or intracellular Ca^2+ ^flux [67]. Shp2's function at the NMJ in vivo also remains elusive, although one study [68] has reported that NMJs appear normal around birth in mice in which Shp2 expression is reduced in muscle. In that study the presence of residual Shp2 in muscle was not ruled out and the formation of pre-patterned AChR clusters ~E14 and the dispersal of these clusters by ~E17 were not examined [68]. Several other findings, however, argue in favor of Shp2's involvement in NMJ development: Shp2 regulates neuregulin/ErbB signaling and AChR gene expression [44], Shp2 limits agrin/MuSK-dependent formation of new AChR clusters [25,43], and Shp2 promotes the disassembly of pre-patterned (this study). Interestingly, Shp2 associates with MuSK and is able to stabilize AChR clusters in rodent myotubes [65], which could be due to its ability to activate src. However, excessive src kinase or tyrosine phosphatase activity also destabilizes AChR clusters in these cells [65], which is compatible with our findings vis-à-vis SIRPα1 and Shp2 and the inhibition of AChR clustering [25,43] and the facilitation of dispersal (this study). Lastly, tyrosine phosphatases have recently also been found to regulate the membrane insertion of AChRs at the NMJ [69] suggesting that additional novel functions of Shp2 and other phosphatase in NMJ development await discovery.

## Conclusion

During the earliest stages of NMJ establishment, motor innervation of muscle induces the clustering of AChRs at incipient synapses as well as the disassembly of pre-patterned AChR aggregates formed in muscle before innervation. In amphibian muscle, synaptogenic stimuli direct both of these processes independently of AChR activity, with the former process being suppressed by tyrosine phosphatases and the latter being mediated by phosphatases. In this study we found that pharmacological or molecular manipulation of one specific phosphatase – Shp2 – produced quantifiable changes in the dispersal of pre-patterned AChR clusters in Xenopus muscle cells: whereas dispersal was blocked by inhibitors of Shp2 signaling, it was accelerated under conditions elevating muscle Shp2 protein or activity levels. Our results suggest a role of Shp2 (and its regulators such as SIRPα1) in not only limiting AChR cluster formation [25,43] but also in promoting the disassembly of pre-patterned AChR clusters by synaptogenic stimuli. Functioning in these two distinct ways Shp2 may enhance the efficiency with which AChRs are concentrated at developing NMJs and depleted from non-synaptic regions of muscle. The participation of other muscle tyrosine phosphatases in the dispersal of pre-patterned AChR clusters, by synaptogenic stimuli or by ACh, and the potential involvement of Shp2 signaling in the dispersal of AChR clusters by ACh remain to be tested.

## Authors' contributions

YKQ, RM and HBP designed the experiments and analyzed the data; YKQ and AWSC carried out the experiments; and YKQ and RM wrote the manuscript. All authors have read and approved the final manuscript.
